# Detecting mass mortality events in wildlife populations

**DOI:** 10.1111/cobi.70136

**Published:** 2025-08-27

**Authors:** Jesse L. Brunner, Justin M. Calabrese

**Affiliations:** ^1^ School of Biological Sciences Washington State University Pullman Washington USA; ^2^ Center for Advanced Systems Understanding (CASUS) Helmholtz‐Zentrum Dresden‐Rossendorf (HZDR) Görlitz Germany; ^3^ Department of Ecological Modelling Helmholtz Centre for Environmental Research‐UFZ Leipzig Germany; ^4^ Department of Biology University of Maryland College Park Maryland USA

**Keywords:** detection probability, die‐off, hidden mortality, mass mortality event, surveillance, evento de mortalidad masiva, extinción, mortalidad oculta, probabilidad de detección, vigilancia, 大规模死亡事件, 大量死亡, 检测概率, 隐性死亡率, 监测

## Abstract

Reports in the literature of mass mortality events (MMEs) involving diverse animal taxa are increasing. Yet, many likely go unobserved due to imperfect detection and infrequent sampling. MMEs involving small, cryptic species, for instance, can be difficult to detect even during the event, and degradation and scavenging of carcasses can make the window for detection very short. Such detection biases make it difficult to understand trends in MMEs across time, regions, or taxa. Thus, we developed a simple modeling framework to clarify key aspects (e.g., sampling frequency, dynamics of detectability) of the problem and spur future work. Our framework describes the probability of detecting an MME as a function of the observation frequency relative to the rate at which MMEs become undetectable. Although simple, this framework is useful for developing an intuition about how the probability of detecting a randomly occurring MME increases with peak detectability, with slower rates of decay in detectability, and with more frequent observations. It can also facilitate the design of surveillance programs. To illustrate its utility, we applied it to *Ranavirus*‐related MMEs in 35 populations of an endangered salamander subspecies. We found that the probability of detecting an MME was <50% and that the frequency of MMEs in this system was likely much greater than the one MME observed in the 35 ponds. The limitations of this framework (e.g., assumption that surveys occur regularly and with equal effort) may help set an agenda for future research in this area.

## Abstract (es)

Detección de eventos de mortalidad masiva en poblaciones silvestres

**Resumen:**

Los informes bibliográficos sobre eventos de mortalidad masiva (EMM) que afectan a diversos taxones animales están aumentando. Sin embargo, es probable que muchos pasen desapercibidos debido a una detección imperfecta y a un muestreo poco frecuente. Por ejemplo, los EMM que afectan a especies pequeñas y crípticas pueden ser difíciles de detectar incluso durante el evento, y la degradación y la descomposición de los cadáveres pueden reducir considerablemente el margen de tiempo para su detección. Estos sesgos en la detección dificultan la comprensión de las tendencias de los EMM a lo largo del tiempo, las regiones o los taxones. Por ello, desarrollamos un marco sencillo de modelación para aclarar los aspectos clave del problema (por ejemplo, la frecuencia de muestreo o la dinámica de la detectabilidad) e impulsar futuros trabajos. Nuestro marco describe la probabilidad de detectar un EMM como una función de la frecuencia de observación en relación con la tasa a la que los EMM se vuelven indetectables. Aunque es sencillo, este marco es útil para desarrollar una intuición sobre cómo la probabilidad de detectar un EMM que se produce aleatoriamente aumenta con el pico de detectabilidad, con tasas más lentas de disminución de la detectabilidad y con observaciones más frecuentes. También puede facilitar el diseño de programas de vigilancia. Para ilustrar su utilidad, lo aplicamos a los EMM relacionados con *Ranavirus* en 35 poblaciones de una subespecie de salamandra en peligro de extinción. Descubrimos que la probabilidad de detectar un EMM era inferior al 50% y que la frecuencia de los EMM en este sistema era probablemente mucho mayor que el único EMM observado en los 35 estanques. Las limitaciones de este marco (p. ej., la suposición de que las encuestas se realizan con regularidad y con el mismo esfuerzo) pueden ayudar a establecer una agenda para futuras investigaciones en este ámbito.

## 野生动物种群中大量死亡事件的检测

**【摘要】** 关于多个动物类群大量死亡事件(mass mortality event, MME)的文献报道正在日益增加。然而, 由于检测不完全和采样频率低, 许多此类事件可能尚未被观察到。例如, 涉及小型隐秘物种的MME即使在事件发生期间也难以检测, 而尸体的降解和清除会使检测窗口非常短暂。此类检测偏差阻碍了对MME在时间、地区及类群间发展趋势的理解。因此, 本研究开发了一个简单的建模框架, 以阐明该问题的关键方面(如采样频率、可检测性动态)并推动未来研究。我们的框架将检测到MME的概率描述为观察频率与MME变得难以检测的速率之比的函数。尽管该框架较为简单, 但它有助于直观理解检测到随机发生的MME的概率如何随可检测性峰值、较慢的可检测性衰减速率及更频繁的采样观察而增加。该框架还有助于监测项目的设计。为展示该框架的应用价值, 我们将其应用于濒危蝾螈亚种35个种群中与蛙病毒(*Ranavirus*)相关的MME检测之中。我们发现, 检测到MME的概率低于50%, 且该系统中MME的发生频率很可能远高于在35个池塘中观察到的单一MME。该框架的局限性(如假设定期且以相同强度进行调查)可能为该领域的未来研究设定议题。**【翻译:胡怡思;审校:聂永刚】**

## INTRODUCTION

Mass mortality events (MMEs), defined as “rapidly occurring catastrophic demographic events that punctuate background mortality levels” (Fey et al., 2015), can have profound impacts on populations, communities, and ecosystems (Lamberti et al., 2020; Tye et al., 2024). Whether local (e.g., bats in hibernacula [Fritze & Puechmaille, 2018]) or widespread (e.g., saiga antelopes across a landscape [Kock et al., 2018]), such rapid demographic losses can frustrate recovery efforts and contribute to further declines of vulnerable populations, making MMEs especially relevant to conservation. The growing number of reported MMEs in invertebrate and vertebrate animal populations suggests an increasing incidence of MMEs, which are often associated with natural and anthropogenic perturbations including disease, toxins, and multiple interacting stressors (Fey et al., 2015; Tye et al., 2022). Although MMEs can produce spectacular levels of mortality, leaving hundreds to hundreds of thousands of carcasses in their wake, many are much smaller in magnitude (Fey et al., 2015). Moreover, carcasses are scavenged, decompose, sink from view, or otherwise disappear (Santos et al., 2016), making MMEs difficult to detect without frequent surveillance (Kennedy et al., 2017; Santos et al., 2016). This is likely especially important for MMEs involving soft‐bodied species in environments with large numbers of scavengers or rapid rates of decay or flow through (Kennedy et al., 2017; Phelps et al., 2019; Santos et al., 2016; Teixeira et al., 2013). And, MMEs involving small, rare, or cryptic species may be difficult to detect even at the peak of an event. Thus, many MMEs likely remain undetected, leading to an inherent negative bias to estimates of MMEs, one that is magnified in certain taxa and settings, making simple extrapolations from reported rates unreliable.

Unfortunately, quantifying detection probabilities and accounting for biases are difficult based on most published accounts. Basic details of how MMEs were detected, even whether they were discovered coincidentally or as part of routine surveillance, are usually absent (Fey et al. [2015] and references therein). The literature, however, does appear biased toward more easily detected species and accessible settings. For instance, half (52%) of the 460 reports used by Fey et al. (2015) involved fishes, perhaps because large numbers of carcasses floating near shore or in fishing areas are difficult to miss. Most of the 64 MMEs of mammals involved large‐bodied species. All but 5 of the 27 MMEs in amphibians involved species associated with water bodies, where MMEs would be easier to detect, and the few reports in terrestrial environments involved intense surveillance efforts (Attademo et al., 2011; Brem & Lips, 2008; Crawford et al., 2010) or well‐observed locations (Duff et al., 2011; Holland et al., 1990). Indeed, outside of systems frequently observed in research or other activities (e.g., hunting or fishing), most reports of MMEs seem to involve a large measure of serendipity.

Although it is apparent that MMEs are detected imperfectly in many contexts and taxa, a coherent framework with which to determine either the magnitude of this bias or the factors that contribute to it is lacking. There is an established literature on detection of carcasses (e.g., during road surveys); however, existing models focus on estimating steady state mortality or kill rates (e.g., Teixeira et al., 2013) and do not extend straightforwardly to detecting MMEs, which are sporadic, intense events. We therefore took the first step by building simple models to describe and explore how the detection of MMEs varies with key quantities, such as peak detectability, rate of change in detectability over time, and frequency of surveillance.

Although the assumptions on which we based our models are likely too restrictive for them to be applied directly to many real systems or settings, the advantage of this simple approach is that it makes clear the importance of these few variables and the relationships between them and should help conservation professionals structure their thinking and develop an intuition for the problem. To this end, we also developed R functions to implement these models, which may also facilitate the design of stronger surveillance efforts for detecting MMEs. The limitations of our current framework (detailed below) should help set an agenda for future research in this area. Finally, although our models were developed in the context of MMEs involving animals, there are no obvious reasons why they could not also be applied to plants.

## METHODS

In some systems, an MME may leave a demographic signature, such as a clear drop in the size of a well‐monitored population, that can be easily distinguished from other causes (e.g., migration or metamorphosis). Here, however, we considered only the probability of detecting an MME directly, by observing dead or dying individuals. Although Fey et al. (2015) provide an operational definition of MMEs as “rapidly occurring catastrophic demographic events that punctuate background mortality levels,” there are no clear threshold levels of mortality for categorizing mortality events as MMEs or something short of that. Moreover, such data are often unavailable. Among the 1183 MMEs Fey et al. (2015) considered, there are estimates of the proportion of the population affected for only 114. The spatial extent of mortality for an event to be described as an MME is also undefined and is based, in practice, on researchers’ knowledge of the taxon, setting, and ecology rather than any particular thresholds or decision rules (e.g., a spatial area containing a given fraction of the population). Although the study of MMEs would benefit from more rigorous definitions, they may be difficult to operationalize in a general way. For our purposes, we simply assumed that researchers consider mortality of an appropriate extent at an appropriate spatial scale for their study systems.

**FIGURE 1:**
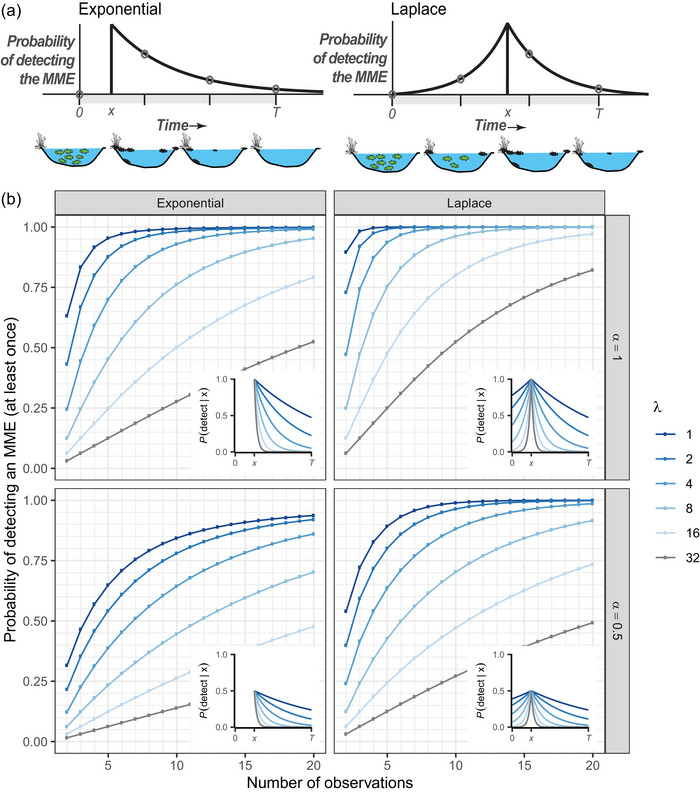
(a) How the probability of detection might change as a function of time from the peak of the mass mortality event (MME) in an exponential (left panel) or Laplace (right panel) model (pond images, state of a population of fish at each of 4 sampling times; green fish, live fish; black fish, carcasses) and (b) the marginal probability of detecting an MME occurring at some random time, x, as a function of the number observations made at regular intervals based on the assumption that the detection probability over time is either exponential (left panel) or Laplace (right panel). Inset graphs show decline in detection probabilities from a maximum of α=1 (top row) or α=0.5 (bottom row) at the peak of the MME at time x given different values of λ (rate of decline relative to the length of the time in which MMEs can occur, T).

We made the simplifying assumption that a single MME occurs randomly, with uniform probability at any time, (0,T), and during a season of length T (Figure 1a). Considering single events avoids complications arising from potential dependence between events in terms of timing or detectability and is realistic in situations where surveillance efforts or MMEs are strongly seasonal or involve such a large fraction of individuals that a subsequent event would be essentially undetectable. We also assumed n observations are made regularly during the period starting at t=0, then at intervals of T/(n−1), and concluding at t=T.

We developed 2 versions of the model to reflect how detection probabilities might change through time (Figure 1a). In the first model, the MME occurred instantaneously and had an initial (peak) detectability α∈[0,1], which then decayed exponentially at rate λ>0 (Figure 1a). Those MMEs that are easily detected by an observer, such as events involving large or abundant species in easily observed locations (e.g., charismatic megafauna in open fields or fishes near shore), would have a large value of α. In contrast, MMEs involving small, cryptic, or rare species in locations that are difficult to study (e.g., small shrews in dense forests or fossorial caecilians) would have small values of α. Similarly, small, soft‐bodied taxa that degrade quickly or that are rapidly scavenged or washed out of aquatic systems (e.g., Phelps et al., 2019) would have larger values of λ, meaning the detection probability decays faster. Although the probability of detecting animal carcasses, and thus MMEs, depends on myriad factors affecting carcass persistence and detectability (e.g., Korner‐Nievergelt et al., 2015; Santos et al., 2016), exponential decay is a reasonable first approximation of the general trend of declining detection probability as carcasses decay or are removed at a constant rate (e.g., Regester & Whiles, 2006).

We based the second model on the assumption that detection probability follows a Laplace (i.e., back‐to‐back exponential) distribution, such that detection probability first increased exponentially at rate λ, peaked at α, and then declined exponentially at rate λ thereafter (Figure 1a). This model may be more representative of autocatalytic events, such as epidemics. In both cases, we used rescaled (to peak at α) probability functions as convenient functional forms that represent change in detection probability versus time during the season and did not assume that detection probabilities are random variables.

For simplicity, we scaled time in both models such that T=1, and we defined λ relative to this period. In addition to eliminating the need to explore how T influences model behavior, this formulation highlighted the intuitive notion that it is not the absolute time between observations, but rather the time between observations, T/(n−1), relative to the rate at which MMEs become undetectable (λ) that determines one's chance of detecting an event. In any case, one can always rescale to an absolute rate (e.g., day^−1^) as $\lambda_{\mathrm{absolute}}=\lambda_{\mathrm{scaled}}/T$, for T>1.

Given this framework, the probability of detecting an MME that occurs at time t=x in the n equally spaced observations was one minus the product of the probabilities of not detecting the event at each observation. However, the event time, x, was a uniformly distributed random variable over the (scaled) season (0,1). Therefore, to obtain the marginal probability of detection, which accounts for variation in event timing, one must integrate over the distribution of x.

The marginal probability of detecting a randomly occurring mortality event is therefore given by

(1) $$P_{n}\left(\mathrm{detect}\;|\;\alpha ,\lambda \right)=1-\int_{x=0}^{x=1}\prod_{i=0}^{n-1}\left[1-\alpha f\left(\frac{i}{n-1}-x\;|\;\lambda \right)\right]d,$$

where for the exponential case we used

(2) $$f_{E}\left(z\;|\;\lambda \right)=\left\{\begin{array}{cc} e^{\lambda \left(-z\right)}, & z\geq 0\\ 0, & z<0 \end{array}\right.,$$

which resulted in the marginal probability $P_{N}^{E}\left(\mathrm{detect}|\alpha ,\lambda \right)$. For the Laplace case, we used

(3) $$f_{L}\left(z\;|\;x,\lambda \right)=\left\{\begin{array}{cc} e^{\lambda \left(x-z\right)}, & z\geq x\\ e^{\lambda \left(z-x\right)}, & z<x \end{array}\right.,$$

which resulted in the marginal probability $P_{N}^{L}\left(\mathrm{detect}|\alpha ,\lambda \right)$. Equations (2) and (3) were based on the probability density functions (PDFs) of the exponential and Laplace distributions, respectively, but were not normalized because we used them as convenient functional forms that peak at one rather than as proper PDFs that integrate to one.

We derived the exact solutions of both the exponential and Laplace models for the n=2 case (Appendix S1). For the exponential model, the solution was

(4) $$P_{2}^{E}\left(\mathrm{detect}\;|\;\alpha ,\lambda \right)=\frac{\alpha }{\lambda }\left(1-e^{-\lambda }\right).$$

Direct inspection of this solution highlights how the probability of detecting an MME increased linearly (and thus monotonically) as α increased and decreased monotonically as λ increased (verified in Appendix S1). The exact solution of the Laplace model for the n=2 case was

(5) $$P_{2}^{L}\left(\mathrm{detect}|\alpha ,\lambda \right)=\frac{\alpha }{\lambda }\left(2-2e^{-\lambda }-\alpha \lambda e^{-\lambda }\right).$$

For the n=2 case of the Laplace model, as with the exponential model, the probability of detecting an MME increased monotonically as α increased and decreased monotonically as λ increased (shown formally in Appendix S1).

Although exact solutions to Equation (1) can be constructed for particular values of n, as shown above and in Appendix S1 for the n=2 case, they become cumbersome for larger n. Instead, the general model (Equation 1) can be solved numerically for any n≥2 and choice of exponential or Laplace functions (see Appendix S2 for R code to implement these models).

## RESULTS

**FIGURE 2:**
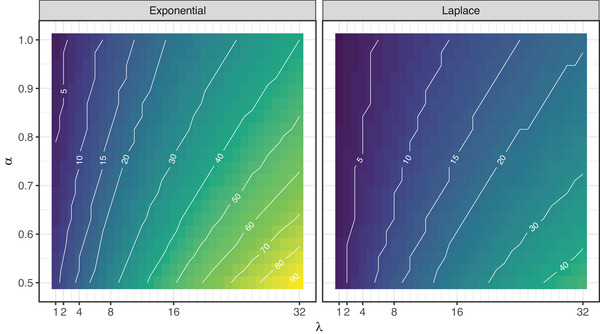
Number of regular observations (n, lines) required to achieve a ≥80% chance of observing a mass mortality event given particular values of the peak detection probability, α, and the rate at which detection probability declines after the event, λ, in the exponential (left panel) and Laplace (right panel) models (white lines, isoclines for particular numbers of observations).

For both models, events that were easily detected at their peak (high values of α) and that remained detectable over long periods (low values of λ) were more likely to be detected than those with low detectability at their peak (low α) or that quickly faded (high values of λ) (Figure 1b). Similarly, events that became more detectable up to the peak of the event and then declined thereafter (Laplace model [Figure 1b]) were, all else being equal, more easily detected than those that became instantly detectable and then declined (exponential model [Figure 1b]). These results also provided numerical evidence that suggested the analytically confirmed monotonic relationships between the probability of detection and both α and λ for the n=2 case also held for larger n (Figure 1). Although these qualitative results were intuitive, it is striking how frequent observations must be to ensure a reasonable probability of detecting rapidly fading events (Figure 2). Five surveys during a season guaranteed a ≥80% chance of detecting an MME for only the 2 lowest decay rates (λ≤2) considered in Figure 1b when the event was perfectly detectable at its peak (α=1). If there were only a 50% chance of detection at the peak (α=0.5), at least twice the number of observations would be required to achieve the same level of confidence for a given λ (Figure 2).

As an illustration of the difficulty detecting MMEs with any confidence, consider J.B.’s experience attempting to find *Ranavirus*‐related MMEs in an endangered subspecies of tiger salamander (*Ambystoma [tigrinum] mavortium stebbinsi* [Irschick & Shaffer, 1997]) inhabiting earthen cattle‐watering tanks in southern Arizona. These events occur suddenly and sporadically at any time of year in this system and can kill most or all aquatic stages. However, salamander larvae also metamorphose and leave, and ponds occasionally dry and refill, meaning the absence of larvae from a tank is not necessarily evidence of an MME. During one intense year of sampling, 35 tanks were seined or observed from shore 3–12 times (mean = 5.26, mode = 4), but just one MME was observed. Is 1 in 35 a reasonable estimate of the frequency of MMEs in this system? How likely were we to have detected MMEs given these frequencies of observations?

We used observations of 11 MMEs in 8 tanks in prior years to estimate λ by fitting the exponential model of detection probability from the start of an MME (= first observation) to the binary detection data at each visit (Appendix S3). This yielded $\lambda_{\mathrm{absolute}}\approx 0.022$ per day and thus $\lambda_{\mathrm{scaled}}=0.022\times 365\approx 8$. We also assumed α=1 because these MMEs were large events in simple habitats free of most vegetation, and carcasses were often found floating or on shore (i.e., a best‐case scenario). The exponential model with λ=8 and α=1 suggested our probability of detecting an MME in a tank varied from $P_{3}^{E}=0.25$ with 3 visits to $P_{12}^{E}=0.83$ with 12 visits (overall average 0.44) (see the line corresponding to λ=8 in the upper left panel of Figure 1b). Among the 16 tanks with 4 visits, the most common situation, there was a good chance that an MME would have gone undetected ($1-P_{4}^{E}=0.64$).

Although these are very rough estimates that do not account for many details of the system and sampling (e.g., visits did not always occur at regular intervals, tanks varied in size and complexity), they suggested the actual frequency of MMEs was likely much >1 in 35 tanks in the survey. So, it was unsurprising that the one MME we detected was in a tank with more visits ($P_{8}^{E}=0.67$). This outcome also suggested that achieving an MME detection probability of 0.8, for example, would require n≥11 observations (Figure 2). If the peak probability of detection were even slightly lower, this number would increase (e.g., with α=0.9, n≥13). Thus, the apparent absence of MMEs in subsequent years of surveillance should be interpreted with caution.

## DISCUSSION

Our exponential model of the probability of detecting MMEs provides a rough approximation for events that occur suddenly, producing a pulse of mortality, the signs of which then decline over time relatively more slowly. Examples might include die‐offs in aquatic systems caused by dead zones or red tides, where mortality occurs rapidly and then carcasses slowly decay. The Laplace model is more appropriate for MMEs that become increasingly severe, or at least become more easily detectable, up to a peak and then wain at approximately the same rate. This model might be useful for describing the detectability of die‐offs due to transmissible disease or caused by, for instance, prolonged heatwaves that kill larger and larger swaths of an increasingly vulnerable population. However, these models are highly simplified and should not be interpreted mechanistically. Rather, they are intended to formalize intuition about how key parameters (e.g., surveillance frequency relative to the rate at which MMEs become undetectable) translate to detection probabilities. They also highlight, as we illustrated in the example with MMEs in tiger salamanders, how difficult it can be to detect MMEs.

The clear conclusion is that surveillance efforts must become more frequent to have a reasonable chance of detecting MMEs in many taxa and settings. When resources do not allow for such intense monitoring by professionals, public engagement and citizen science programs may be an effective way to increase the number of observations. Public awareness campaigns and efforts to streamline reporting to appropriate officials can also be thought of as ways of improving the chance of MME detection. However, public reports should be followed up by professionals so that events can be appropriately classified as MMEs.

Currently few, if any, published accounts of MMEs report key factors that might help readers even informally correct for detection probability (i.e., α, λ, and n). At a minimum, reporting of the sampling frequency (n), relevant details of the focal area, and search effort in the study should be encouraged. It may also be possible to estimate detection probabilities at the peak of an event (α) and the decay in detectability over time (λ) with experiments or observational studies in which surveys are repeated over time (e.g., Kennedy et al., 2017) or replicate observers are used (e.g., Santos et al., 2016). Even coarse estimates, such as those we present for the *Ranavirus*–tiger salamander system, would allow researchers to identify important biases in observed rates of MMEs and design stronger surveillance programs. They would also allow conservation programs to better consider the impact of MMEs on their efforts. We also encourage reporting of the apparent consequences and spatial extents of MMEs so that events and their impacts can be better characterized.

Our models are necessarily simple and built around strong assumptions that prevent their direct application to many systems and settings. For instance, rather than standardized surveillance at regular intervals, detection of MMEs in many systems likely comes from sporadic visits with unequal efforts. It would be useful to expand our approach to accommodate such irregular observation intervals, covariates that modify detection probabilities (e.g., search intensity, time of year, time‐dependent population sizes), and, potentially, multiple MMEs within a season. Moreover, although we used exponential and Laplace functions to describe how detectability changes through time, it should be relatively straightforward to modify our model with other functional forms that better describe patterns in the detectability of MMEs. It is also possible to explicitly model the dynamics of carcass production and loss (e.g., during an epidemic) and their relationship to detection probabilities, but this would require a different, dynamic framework (e.g., a compartment model) and would necessarily be system specific.

We hope this work provides impetus for further empirical studies on how detection probabilities vary through time and among settings. Our initial work, however, suggests that the true rates of MMEs are likely substantially higher than what is observed—especially, based on first principles, for small species in hot, difficult‐to‐study locations, where much of the world's biodiversity resides.

## AUTHOR CONTRIBUTIONS

**Jesse L. Brunner**: Conception; resources; data curation; visualization; writing—original draft; writing—revision and editing. **Justin M. Calabrese**: Conception; methodology; formal analysis; software; writing—revision and editing.

## Supporting information



Supporting information

Supporting information
