# Supplementary material for: Detecting mass mortality events in wildlife populations
Source: Conserv Biol. 2025 Aug 27;40(1):e70136. doi: 10.1111/cobi.70136 (PMC12856798; doi:10.1111/cobi.70136)
Supplement: Supplementary file 1 — Supporting information [file COBI-40-e70136-s001.pdf]

# Appendix S1 for: The problem of detecting mass mortality events in wildlife populations

In this appendix, we derive and analyze closed-form solutions for the case of two observations per season under both the exponential and Laplace models of MME detection.

## A Exponential model

For the exponential case, we substitute $n = 2$ and eqn (2) into eqn (1) and simplify to obtain

$$P_2^E(\text{detect}|\alpha, \lambda) = 1 - \int_0^1 (1 - \alpha \mathrm{e}^{-(1-x)\lambda})\, dx \tag{A1}$$

$$= \alpha \int_0^1 \mathrm{e}^{-(1-x)\lambda}\, dx, \tag{A2}$$

where $\alpha$ and $\lambda$ are the peak detection probability and the scaled decay rate, respectively. Notice that the $i = 0$ term in the product of eqn (A1) always evaluates to 1 and is therefore ignored here. We then solve the integral in eqn (A2) via u-substitution. Specifically, we let $u = -(1 - x)\lambda$, $dx = du/\lambda$, and rewrite the limits of integration in terms of $u$, which yields

$$P_2^E(\text{detect}|\alpha, \lambda) = \frac{\alpha}{\lambda} \int_{-\lambda}^0 \mathrm{e}^u\, du. \tag{A3}$$

Finally, using the fact that the antiderivative of $\int \mathrm{e}^u$ is $\mathrm{e}^u$, we evaluate the antiderivative on $[-\lambda, 0]$ and simplify to obtain

$$P_2^E(\text{detect}|\alpha, \lambda) = \frac{\alpha}{\lambda} \mathrm{e}^u \Big|_{-\lambda}^0 \tag{A4}$$

$$= \frac{\alpha}{\lambda}(1 - \mathrm{e}^{-\lambda}), \tag{A5}$$

which is eqn (4) in the main text. It is clear from direct inspection that this function increases linearly (and thus monotonically) in $\alpha$, which makes intuitive sense. To verify that eqn (A5) decreases monotonically in $\lambda$, we take its derivative with respect to $\lambda$

$$\frac{dP_2^E}{d\lambda} = \frac{\alpha \mathrm{e}^{-\lambda}}{\lambda^2}([1+\lambda] - \mathrm{e}^{\lambda}), \tag{A6}$$

where we have dropped the dependence of $P_2^E$ on the parameters for notational convenience. The term in parentheses, $[1+\lambda]-\mathrm{e}^{\lambda}$, will determine the sign of the derivative. Expanding the exponential function in this term to first order in $\lambda$ yields

$$1 + \lambda + \mathcal{O}(\lambda)^2 > 1 + \lambda, \tag{A7}$$

which means that $\frac{dP_2^E}{d\lambda}$ is always negative, and thus $P_2^E$ decreases monotonically in $\lambda$.

# B Laplace model

For the Laplace model, we substitute eqn (3) into eqn (1) and let $n = 2$. Noting the limits of integration and simplying, we have

$$P_2^L(\text{detect}|\alpha, \lambda) = 1 - \int_0^1 (1 - \alpha \mathrm{e}^{-\lambda x})(1 - \alpha \mathrm{e}^{\lambda(x-1)})\, dx, \tag{B1}$$

where $\alpha$ and $\lambda$ are as before, and unlike the case with the exponential model, the $i = 0$ term of the product in the Laplace model is not equal to 1 and so is retained. Expanding the product in the integrand and integrating term-by-term, we obtain

$$P_2^L(\text{detect}|\alpha, \lambda) = 1 - (\int_0^1 1\, dx - \int_0^1 \alpha \mathrm{e}^{\lambda(x-1)}\, dx - \int_0^1 \alpha \mathrm{e}^{-\lambda x}\, dx + \int_0^1 \alpha^2 \mathrm{e}^{-\lambda}\, dx) \tag{B2}$$

$$= 1 - (1 - \alpha \int_0^1 \mathrm{e}^{\lambda(x-1)}\, dx - \alpha \int_0^1 \mathrm{e}^{-\lambda x}\, dx + \alpha^2 \mathrm{e}^{-\lambda}), \tag{B3}$$

where the second line follows as the first and last integral terms are just constants. We tackle the remaining two integral terms via $u$-substitution. Let $u_1 = \lambda(x-1)$, $dx_1 = (1/\lambda)du_1$, $u_2 = -\lambda x$, and $dx_2 = (-1/\lambda)du_2$. Next, we rewrite the limits of integration in terms of $u_1$ for the first integral term and $u_2$ for the second, which yields

$$P_2^L(\text{detect}|\alpha, \lambda) = 1 - (1 - \frac{\alpha}{\lambda} \int_{-\lambda}^0 \mathrm{e}^{u_1}\, du_1 + \frac{\alpha}{\lambda} \int_0^{-\lambda} \mathrm{e}^{u_2}\, du_2 + \alpha^2 \mathrm{e}^{-\lambda}). \tag{B4}$$

Again using the fact that the antiderivative of $\int \mathrm{e}^u$ is $\mathrm{e}^u$, we evaluate each of the antiderivatives over their respective limits of integration and simplify to obtain

$$P_2^L(\text{detect}|\alpha,\lambda) = 1 - (1 - \frac{\alpha}{\lambda}\mathrm{e}^{u_1}\Big|_{-\lambda}^{0} + \frac{\alpha}{\lambda}\mathrm{e}^{u_2}\Big|_{0}^{-\lambda} + \alpha^2\mathrm{e}^{-\lambda}) \tag{B5}$$

$$= 1 - (1 - \frac{\alpha}{\lambda}(1-\mathrm{e}^{-\lambda}) - \frac{\alpha}{\lambda}(1-\mathrm{e}^{-\lambda}) + \alpha^2\mathrm{e}^{-\lambda}) \tag{B6}$$

$$= \frac{\alpha}{\lambda}(2 - 2\mathrm{e}^{-\lambda} - \alpha\lambda\mathrm{e}^{-\lambda}), \tag{B7}$$

which is eqn (5) in the main text.

To establish that eqn (B7) increases monotonically in $\alpha$ and decreases monotonically in $\lambda$, we consider its derivatives first with respect to $\alpha$, and then with respect to $\lambda$. Differentiating eqn (B7) with respect to $\alpha$ yields

$$\frac{dP_2^L}{d\alpha} = \frac{2\mathrm{e}^{-\lambda}}{\lambda}(\mathrm{e}^{\lambda} - \alpha\lambda - 1) \tag{B8}$$

$$= \frac{2\mathrm{e}^{-\lambda}}{\lambda}(\mathrm{e}^{\lambda} - [\alpha\lambda + 1]), \tag{B9}$$

where we have again dropped the parameter dependence of $P_2^L$. We again note that the term in parentheses will determine the sign of the derivative. We now set $\alpha = 1$ noting that the right-hand component of this term, $-[\alpha\lambda+1]$ will attain its most negative value in this case. Finally, expanding the exponential to first order in $\lambda$, we have

$$1 + \lambda + \mathcal{O}(\lambda)^2 > 1 + \lambda, \tag{B10}$$

which implies that $\frac{dP_2^L}{d\alpha}$ will always be positive, and thus $P_2^L$ increases monotonically in $\alpha$.

Next, differentiating eqn (B7) with respect to $\lambda$ we obtain

$$\frac{dP_2^L}{d\lambda} = \frac{\alpha\mathrm{e}^{-\lambda}}{\lambda^2}(2 + \lambda(2 + \alpha\lambda) - 2\mathrm{e}^{\lambda}) \tag{B11}$$

$$= \frac{\alpha\mathrm{e}^{-\lambda}}{\lambda^2}([2 + 2\lambda + \alpha\lambda^2] - 2\mathrm{e}^{\lambda}). \tag{B12}$$

Again, the term in parentheses will determine the sign of the derivative, and the term in square brackets will attain its largest value when $\alpha = 1$, so we focus on this case. Expanding the function $2\mathrm{e}^{\lambda}$ to second order in $\lambda$ yields

$$2 + 2\lambda + \lambda^2 + \mathcal{O}(\lambda)^3 > 2 + 2\lambda + \lambda^2, \tag{B13}$$

which implies that $\frac{dP_2^L}{d\lambda}$ is always negative and thus that $P_2^L$ decreases monotonically in $\lambda$.
