# Supplementary material for: Detecting mass mortality events in wildlife populations
Source: Conserv Biol. 2025 Aug 27;40(1):e70136. doi: 10.1111/cobi.70136 (PMC12856798; doi:10.1111/cobi.70136)
Supplement: Supplementary file 2 — Supporting information [file COBI-40-e70136-s002.pdf]

Appendices S2 & S3 for

Hidden die-offs: How likely are we to detect mass mortality events?

Appendix S2

Here we present an R function to numerically solve equations (1) or (3) for any $n \geq 2$. We first create an un-normalized Laplace probability density function with location parameter, `mu`, and the exponential growth and decay rate, `lambda`.

```
dlpl <- function(x, mu, lambda) exp(-abs(x-mu)*lambda)
```

Next, we create a function that returns the numerical solution from the probability of detecting an MME given either the exponential model (`mod=Exp`) or the Laplace model (`mod=Lpl`). The parameter `lambda` must be greater than zero, `alpha` must be between zero and one, and `n` should be an integer ≥ 2. The function first creates an integrand function of the probability of not detecting an MME that is then integrated and subtrated from one to produce the probability of observing the MME at least once.

```
pDetect <- function(lambda, alpha, n, mod='Exp'){

  #check parameter values and warn appropriately.
  if(n != round(n)) warning("n must be an integer")
  if(n < 2) warning("n must be >=2")
  if(lambda <= 0) warning("lambda must be >0")
  if(alpha <0 | alpha >1) warning("alpha must be >=0 and <=1")

  n <- n - 1

  #build the integrand based on the parameters.
  integrand <- function(lambda, alpha, n, mod){
    function(x){
      int <- dunif(x)
      if(mod=='Exp'){
        for(i in 0:n) int <- int * (1 - (alpha/lambda)*dexp((i/n)-x, lambda))
      } else if(mod=='Lpl'){
        for(i in 0:n) int <- int * (1 - alpha*dlpl(x, i/n, lambda))
      } else{
        warning("mod must be either 'Exp' or 'Lpl'")
      }
      return(int)
    }
  }

  #evaluate the numerical integral and return prob of dectection.
  return(1 - integrate(integrand(lambda, alpha, n, mod), lower=0, upper=1, subdiv
isions = 1000L)$value)
}
```

Appendix S3

Here we provide the data from the example presented in the main text on *Ranavirus*-related mass mortality events (MMEs) in tiger salamanders (*Ambystoma mavortium* [*tigrinum*] *stebbinsi*) and the code to estimate λ, the rate at which the MMEs became undetectable. These data consist of 11 MMEs observed in eight cattle watering tanks during several bouts of semi-regular visits that took place from 1985 through 1998 in the San Rafael Valley in southern Arizona, U.S.A by members of James P. Collins' laboratory group. The intensity of sampling varied, but in each site visit the tank would be seined at least once and at least part of the pond perimeter would be surveyed. For our purposes, we considered a mortality event any site visit in which researchers observed obviously diseased (hemorrhaging, bleeding, edema) or dead larvae or branchiate or metamorphs for which other causes could not be determined (e.g., injury, predation). In our experience in this system and elsewhere carcasses disappear (e.g., are scavenged; Le Sage et al., 2019) rapidly and so the carcasses likely represent recent mortality. Observations of mortality or disease were incidental to the purposes of the tanks visits during this period and thus reflect a background probability of detection during normal research in this system Finally, we cannot be certain of the start of the events, so we set the first observation of the MME as day zero.

```
SRV <- data.frame(Site = c("A", "A", "A", "A", "A", "A", "A",
                           "B", "B", "B", "B", "B", "B", "B",
                           "C", "C", "C", "C", "C",
                           "D", "D", "D", "D", "D",
                           "E", "E", "E", "E",
                           "F", "F", "F", "F", "F", "F", "F",
                           "G", "G", "G", "G",
                           "H", "H", "H", "H", "H", "H", "H"),
                  # Days from when mortality was first noted
                  Days = c(0, 18, 54, 77, 0, 27, 45,
                           0, 18, 54, 77, 115, 126, 173,
                           0, 33, 79, 0, 200,
                           0, 33, 230, 0, 270,
                           0, 22, 69, 105,
                           0, 83, 128, 0, 25, 191, 277,
                           0, 11, 58, 128,
                           0, 18, 54, 77, 104, 173, 209),
                  # Mortality noted (1) or not (0)
                  Mort = c(1, 1, 0, 0, 1, 0, 0,
                           1, 1, 1, 1, 1, 0, 0,
                           1, 0, 0, 1, 0,
                           1, 0, 0, 1, 0,
                           1, 0, 0, 0,
                           1, 0, 0, 1, 0, 0, 0,
                           1, 1, 0, 0,
                           1, 1, 0, 1, 0, 0, 0)
)
```

We fit a model in which the probability of detecting mortality in a tank declined exponentially with time since the first detection. We fit the model to all tanks together, using the maximum likelihood maximizing `mle2` function in the `bbmle` package in R (Bolker and R

Development Core Team, 2022), to generate an estimate for the collection of tanks in the area.

```
library(bbmle)
mle2(Mort ~ dbinom(size=1, prob = exp(-rate*Days)),
     start=list(rate=1),
     data=SRV)
```

This yields and estimate of yielded $\lambda_{\text{absolute}}$ (`rate`) = 0.022 per day (95% CI = 0.013 – 0.036; Figure S1). We assume that MMEs can occur any time in the year in this area, which is reflected in the historical data of observed mortality events, and so we scale this rate to a year as $\lambda_{\text{absolute}} \times 365$ = 8.133 (95% CI = 4.915 – 13.03).

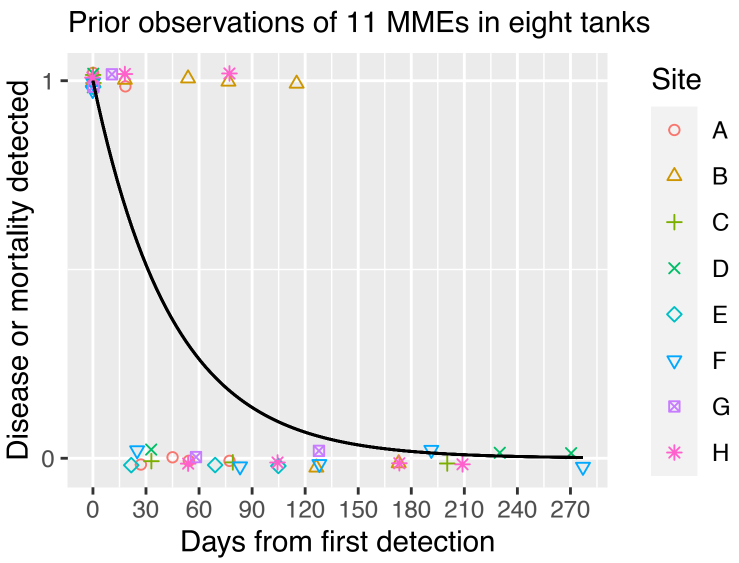

Figure S1: Maximum-likelihood estimate of the probability of detecting disease or mortality, attributed to a MME, over time from first detection (black line). Points represent visits to each earthen cattle tank (A–H), jittered in the y-direction to avoid overplotting.

The probability of detecting a MME declines by half over the first 30 days from an event. This may due to event endings (e.g., most or all individuals are dead or the epidemic has burned out) or simply reduced capacity to detect an ongoing event as fewer individuals are involved.

References cited

Bolker, Ben and R Development Core Team. 2022. “bbmle: Tools for general maximum likelihood estimation.” http://CRAN.R-project.org/package=bbmle

Le Sage, Mitchell J., Bailey D. Towey, and Jesse L. Brunner. 2019. “Do scavengers prevent or promote disease transmission? The effect of invertebrate scavenging on *Ranavirus* transmission.” *Functional Ecology* 33 (7):1342-1350.
